# Viper bites complicate chronic agrochemical nephropathy in rural Sri Lanka

**DOI:** 10.1186/1678-9199-20-33

**Published:** 2014-08-11

**Authors:** Anjana Silva, Rivikelum Samarasinghe, Senaka Pilapitiya, Niroshana Dahanayake, Sisira Siribaddana

**Affiliations:** 1 Department of Parasitology, Faculty of Medicine and Allied Sciences, Rajarata University of Sri Lanka, Saliyapura 50008, Sri Lanka; 2 Department of Community Medicine, Faculty of Medicine and Allied Sciences, Rajarata University of Sri Lanka, Saliyapura, Sri Lanka; 3 Department of Medicine, Faculty of Medicine and Allied Sciences, Rajarata University of Sri Lanka, Saliyapura, Sri Lanka

**Keywords:** Snakebite, Chronic kidney disease, Agricultural nephropathy, Epidemiology, Anuria

## Abstract

Snakebite is a common occupational health hazard among Sri Lankan agricultural workers, particularly in the North Central Province. Viperine snakes, mainly Russell’s viper envenomation, frequently lead to acute renal failure. During the last two decades, an agrochemical nephropathy, a chronic tubulointerstitial disease has rapidly spread over this area leading to high morbidity and mortality. Most of the epidemiological characteristics of these two conditions overlap, increasing the chances of co-occurrence. Herein, we describe four representative cases of viperine snakebites leading to variable clinical presentations, in patients with chronic agrochemical nephropathy, including two patients presented with acute and delayed anuria. These cases suggest the possibility of unusual manifestations of snakebite in patients with Sri Lankan agrochemical nephropathy, of which the clinicians should be aware. It could be postulated that the existing scenario in the Central America could also lead to similar clinical presentations.

## Background

Sri Lanka has high snakebite related morbidity with 37,000 hospital admissions annually [1]. In the country, snakebite is an occupational hazard associated with agriculture, mostly affecting male farmers [2]. The highest snakebite incidence, mortality and case fatality rates are recorded in the dry zone, (annual rainfall < 2000 mm) particularly in the North Central Province (NCP) where farms (Chena), paddy fields and jungles are in close proximity [1,2]. Russell’s viper (*Daboia russelli*) causes 50% of all snakebites in the dry zone, most of them result in life threatening systemic envenomation characterized by coagulopathy, acute renal failure (ARF) and neurological involvement [1,3,4]. Similarly, Merrem’s hump-nosed pit viper (*Hypnale hypnale*) envenomation may also lead to renal failure [5].

During the last two decades, a chronic kidney disease (CKD) of epidemic proportions disease has been occurring in some areas of NCP and dry zone of Sri Lanka. In a survey of patients that attended the Teaching Hospital Anuradhapura (THA), the main tertiary care center in NCP, 82% of them did not have an identifiable cause for CKD [6]. The disease had a distinctive epidemiology and affected mainly male paddy farmers that drank water from wells in limited geographical and familial clusters [7]. Pathological findings included progressive tubulointerstitial nephropathy, which indicates environmental or occupational agents [8]. Due to the persistent inability to find a cause for it, this disease was called CKD of unknown etiology. Recently, considering the emerging evidences of agrochemical use as the most possible etiology, it was proposed that the condition should be renamed chronic agrochemical nephropathy (CAN).

At THA, there was a 227% increase in live discharges and 354% increase in deaths from 1992 to 2007 among patients with CAN [9]. At present, CAN is the leading cause of death in NCP and its unique distribution suggests an environmental etiology [7,9]. *Itai-itai* disease due to cadmium poisoning and Balkan nephropathy provoked by exposure to aristolochic acid are two common epidemiologically related kidney diseases due to environmental toxins.

The following factors were associated with CAN: consumption of well water, paddy farming, use of pesticides and past history of snakebite [10,11]. Association of heavy metal poisoning, predominately arsenic, with CAN was recently reported [12] and confirmed by the World Health Organization [13]. The above hypothesis relates CAN with contaminated fertilizers and pesticides, which motivated to classify the condition as an agricultural nephropathy.

Unfortunately, CAN shares most of its epidemiological characteristics with snakebite, making rural agricultural communities in NCP of Sri Lanka vulnerable for both these life-threatening conditions. Herein, we report four victims of viperine snakebites aggravated by existing CAN in male farmers, leading to renal complications.

## Case presentations

### Patient 1

A 72-year-old male farmer diagnosed with CAN grade II was bitten on his right ring finger, while he was hand-harvesting paddy at 4:30 p.m. on February 20, 2012. After the bite, the patient was admitted to the local hospital. He took with him the dead snake, which was identified as hump-nosed pit viper (*Hypnale hypnale*) by the attending medical officer. The victim developed pain in the bite site and swelling over the right ring finger. He had no neurological signs or evidence of spontaneous bleeding. The whole blood clotting time (WBCT) on admission and six hours after had been less than 20 minutes (<20 minutes considered to be normal). Indian polyvalent anti-venom (IPA) was not administered.

Since the patient had not passed urine since the snakebite, he was transferred to the Professorial Medical Unit of THA seven hours after the initial admission to the local hospital. On admission to the THA, the patient complained of lower abdominal pain, backache, nausea and several episodes of vomiting. His pulse rate was 80 beats per minute and the blood pressure was 160/90 mmHg. The lungs were clear to auscultation and he had no neurological abnormalities. The patient was catheterized but no urine was seen. His prothrombin time (PT) was 60 s and international normalization ratio (INR) was 1.5. The blood urea nitrogen (BUN), serum creatinine (sCr), Na^+^ and K^+^ were 56 mmol/L, 380 μmol/L, 133 mEq/L, and 4.3 mEq/L respectively. His electrocardiogram was normal. Despite intravenous (IV) injection of 120 mg of furosemide, the patient remained anuric. His blood pressure, six hours after the admission to THA, was 170/90 mmHg, pulse rate was 120 min^−1^ and was having bi-basal crepitations of lungs.

The ultrasound scan of the kidneys showed increased cortical echogenicity and unclear corticomedullary differentiation. Oral furosemide, diltiazem, IV penicillin and cloxacillin were administered. The IV and oral fluid intake was restricted and peritoneal dialysis was initiated on the next day. Following 40 cycles of dialysis, five days after the snakebite, the patient started passing water. Despite further 28 cycles of peritoneal dialysis during the next five days, his BUN and sCr levels remained elevated above 100 mmol/L and 800 μmol/L. His systolic and diastolic blood pressure values remained above 150 and 90 mmHg respectively. One cycle of hemodyalisis was performed on the 12^th^ day following the bite. His urine output increased to over 500 mL/day with gradual decline of BUN and sCr levels and the patient was discharged on 15^th^ day following the snakebite.

### Patient 2

A 56-year-old male farmer, a known patient with CAN undergoing treatment, was admitted to THA on September 7, 2012, following a Russell’s viper (*Daboia russelli*) bite on his left foot. The patient was treated with ten vials of IPA for coagulopathy, which was detected by prolonged WBCT, augmented prothrombrin time and INR. The coagulopathy improved with IPA. He had no clinical or biochemical evidence suggestive of ARF and had mild local swelling. He was discharged from the ward on the fourth day, and his level of sCr on that occasion was 124 μmol/L.

Three weeks later, on October 2, 2012, the patient was admitted to the local hospital complaining of shortness of breath and anuria for five days. He was immediately transferred to THA and was admitted to the Emergency Treatment Unit. On admission, the patient was conscious, dyspneic, pale and had bilateral leg edema. His pulse was 84 bpm and blood pressure was 180/110 mmHg. The peripheral oxygen saturation was 91% and auscultation of chest revealed bibasal crepitations and rhonchi. Although immediately catheterized, no urine was seen. The ECG was normal. Hemoglobin level was 7.8 g/dL, pH 7.37 and arterial oxygen partial pressure was 89 mmHg, while serum K^+^, Na^+^, and blood urea were 6.0 mEq/L, 118 mEq/ L and 24.9 mmol/L, respectively. The patient received respiratory support with 40% oxygen therapy and was transferred to the Medical Intensive Care Unit where he was treated with IV dextrose insulin and calcium gluconate for hyperkalemia.

On the following day, his blood pressure was 130/70 mmHg, whilst hemoglobin, BUN, sCr, Na^+^ and K^+^ levels were 7.9 g/dL, 31.2 mmol/L, 882 μmol/L, 123 mEq/L and 6.6 mEq/L, respectively. The chest radiograph showed bilateral pleural effusions. Peritoneal dialysis was initiated and continued for 56 cycles over the next two days. He was transfused with four units of blood. The hyperkalemia and hyponatremia resolved gradually. Following peritoneal dialysis, his BUN, sCr, Na^+^ and K^+^ levels became 24.6 mmol/L, 640 μmol/L, 126 mEq/L, and 3.9 mEq/L. On the fifth day following admission, patient passed urine. However, on the following day, while aspirating the pleural effusion he developed cardiac arrest and died.

### Patient 3

A 53-year-old male farmer was admitted to a rural hospital, due to a Russell’s viper bite on his right foot, at 7:30 p.m., April 9, 2013. Since the WBCT of the victim was more than 20 minutes, he was immediately transferred to THA. On admission to the Emergency Treatment Unit of THA, at 8:30 p.m., the patient had pain, hemorrhage from the bite site, nausea, swelling, and blurred vision, without any evidence of neurotoxicity. His pulse rate was 60 bpm and the blood pressure was 120/70 mmHg. The WBCT was normal, but as the laboratory clotting time was 13 minutes, 20 vials of IPA were administered intravenously. His total white cell count was 13600/uL, of which 71% were granulocytes. The hemoglobin level was 10.8 g/dL and the sCr, blood urea, Na^+^ and K^+^ levels were 288 μmol/L, 11 mmol/L, 141 meq/L and 3.0 meq/L, respectively. During the next two hours, the patient’s urine output was 150 mL. He had no past history of hypertension or diabetes mellitus. Three hours later, the patient developed ptosis, ophthalmoplegia, dysphagia and muscle tenderness. He had no difficulty in breathing. The urine output during the first twelve hours of hospital stay was 1600 mL. At 10:00 a.m. on the next day, his blood pressure was 140/90 mmHg. He had no gross hematuria, normal WBCT and laboratory clotting time was ten minutes. His urinalysis revealed albuminuria and 50 to 60 red blood cells per high power field. The serum K^+^ level was 8.7 mEeq/L and Na^+^ level was 140 mEq/L.

Following IV calcium gluconate therapy and dextrose insulin, his serum K^+^ level decreased to 3.0 mEq/L. The repeated sCr and BUN levels were 325 μmol/L and 15 mmol/L, respectively. His urine output for the next 24 hours was 1650 mL. On the third day of the hospital stay, the sCr and BUN were 310 μmol/L and 15 mmol/L, respectively. The ultrasound scan showed bilateral contracted kidneys (right kidney pole to pole: 72 mm and left kidney: 77 mm), with blurred corticomedullary differentiation. His blood pressure was 150/100 mmHg and the serum electrolyte levels remained within normal range and urine output was 1750 mL for next 24 hours. The patient was discharged with antihypertensive treatment.

### Patient 4

A 48-year-old male farmer bitten on his right ankle by a Russell’s viper on his farm was admitted to the local hospital within ten minutes. Since he had abdominal pain and blurred vision, he was transferred to THA within one hour. On admission at THA, he had mild pain and swelling over his right ankle, headache, nausea and abdominal pain. His WBCT, PT, INR, activated partial thromboplastin time (APTT), white blood cell count (WBC), sCr, BUN on admission were: more than 20 minutes, 19.5 s, 1.7, 22.75 s, 13000 μL^−1^, 334 μmol/L, and 13.6 mmol/L, respectively. His serum Na^+^ and K^+^ levels were within normal range. The patient developed bilateral ptosis, diplopia, and external ophthalmoplegia rapidly within an hour and was treated with 20 vials of IPA. Six hours later, his clotting profile was normal. However, his sCr and BUN remained elevated (368 μmol/L and 17.9 mmol/L, respectively) after 24 hours, with normal serum Na^+^ and K^+^ levels. He was normotensive and had normal urinary output and blood glucose levels throughout the hospital stay. The ultrasound scan of both kidneys revealed contracted kidneys with blurred corticomedullary differentiation, indicating chronic kidney disease. The features of neuromuscular paralysis were gradually settled on the fourth day of hospital stay and the patient was discharged from the ward with a clinic follow-up plan for CKD.

## Discussion

The four cases presented herein show that viperine snakebite in a CAN patient may lead to a spectrum of clinical features related to the urinary system, ranging from prolonged anuria to complete normality. The manifestations of the first and second patients included prolonged anuria, lasting more than four days. Although oliguria and anuria are not uncommon presentations of ARF after a snakebite, reports on prolonged anuria are infrequent, apart from a patient bitten by a hump-nosed pit viper who had 16 days of anuria [14]. Interestingly, anuria was present immediately after the bite in the first patient. In the second patient, no biochemical or clinical evidence of an ARF was observed during the initial hospital stay, but manifested three weeks following the bite, indicating a delayed renal injury. In the first patient, prolonged peritoneal dialysis was required to reverse the ARF as the patient had grade two chronic renal failure. In addition, there was mild coagulopathy, compared with severe renal insult.

Although not previously diagnosed, bilateral shrunken kidneys and very high sCr levels on admission, along with the risk factors of being a male farmer suggested that the third patient was suffering from CAN. Interestingly, this patient developed transient hyperkalemia and hypertension and his urine output remained normal without progressing to ARF. However, despite having elevated sCr and BUN levels together with coagulopathy and neurotoxicity, the fourth patient did not show any abnormal clinical parameter in the favor of ARF. These different presentations may indicate unpredictability of the renal manifestations in CAN patients after viperine snakebite.

Tubulointerstitial nephritis is considered the major pathological event in CAN. In addition, glomerular sclerosis and vascular changes have also been observed [8]. Viperine venoms are known to cause a varying degree of tubular cell damage throughout the renal tubule and glomerular implications, including glomerulonephritis and vascular changes due to both indirect and direct toxic effects [15]. Tubular and glomerular injuries due to viperine venoms were observed in *in vitro*, *in vivo* and clinical studies [15-17]. Therefore, it is likely that these venoms cause further damage to already injured glomerular, tubular and vascular structures of kidneys of CAN patients, further compromising their renal function. Low hemoglobin levels observed in first and second patients could be directly related to existing CAN.

As shown by several previous studies, CAN is a male predominate disease [6,9,10]. All four patients in this study were male farmers, residing in NCP. Although exact prevalence of CAN is unknown, a recent report of the World Health Organization revealed that 15% of the 15 to 70 years old population in NCP are affected by CAN [13]. Unfortunately, the vast majority of CAN patients are late presenters at advanced stages of the disease, due to the insidious onset and poor socioeconomic status of the patients [11]. Therefore, it is possible to have a large number of undiagnosed CAN patients in the community. The only available antivenom for snakebite victims in Sri Lanka is Indian polyvalent antivenom (IPA), which is raised against Russell’s viper, saw-scaled viper (*Echis carinatus*), Indian krait (*Bungarus caeruleus*) and common cobra (*Naja naja naja*). IPA is not effective in treating hump-nosed pit viper envenomation [18,19]. The poor efficacy of IPA against victims of Sri Lankan Russell’s viper bite is well-documented [20]. The first patient did not receive IPA and although the second patient did, presumably, neutralization of the Russell’s viper venom by IPA may have been ineffective. Therefore, unavailability of safe and effective antivenom to be used against all medically important venomous snakes in Sri Lanka has contributed partly to the complications in both these patients.

During the last two decades, Mesoamerican nephropathy, a chronic kidney disease of uncertain etiology, has rapidly spread over Nicaragua, El Salvador and Costa Rica. Young and middle aged male agricultural workers are the most affected group and many afflicted people are not diagnosed until they reach advanced stage of the disease [21]. Viperine bites are common in this region with 50-80% of all snakebites being due to terciopelo (*Bothrops asper*). Most terciopelo victims are young male agricultural workers and acute kidney injury has been reported in 11-17% of them [22]. Hence, it could be assumed that similar unpredicted clinical presentations and renal outcomes could be expected in snakebite victims in the endemic areas of Mesoamerican nephropathy in Central America.

## Conclusion

Snake envenomation in patients with CAN may precipitate ARF on already existing renal failure, and may lead to unexpected presentations such as immediate or delayed anuria.

## Ethics committee approval

This study was approved by the Ethics Review Committee of the Rajarata University of Sri Lanka.

## Consent

Written informed consent was obtained from the patients 1, 3 and 4, and from the wife of patient 2 for publication of this case report.

## Competing interests

The authors declare that there are no competing interests.

## Authors’ contributions

AS and SS designed the study. AS, RS, ND and SP collected data. SS, SP and ND managed patients. AS drafted the paper. All authors read and approved the final version of the paper.
